# Bevacizumab attenuates osteosarcoma angiogenesis by suppressing MIAT encapsulated by serum-derived extracellular vesicles and facilitating miR-613-mediated GPR158 inhibition

**DOI:** 10.1038/s41419-022-04620-3

**Published:** 2022-03-28

**Authors:** Bao-Dong Wang, Xiao-Jun Yu, Ji-Chun Hou, Bo Fu, Hao Zheng, Qi-Kun Liu, Shan-Xi Wang, Zheng-Gang Bi, Yang Cao

**Affiliations:** 1 Department of Orthopedics, the First Affiliated Hospital of Harbin Medical University, Harbin, 150001 P. R. China; 2 Key Laboratory of Hepatosplenic Surgery, Ministry of Education, Harbin, 150001 P. R. China; 3 Department of Orthopedics, Tongji Hospital, Tongji Medical College, Huazhong University of Science and Technology, Wuhan, 430030 P. R. China; 4 Department of Emergency Surgery, the First Affiliated Hospital of Jiamusi University, Jiamusi, 154000 P. R. China

**Keywords:** Tumour angiogenesis, Tumour angiogenesis

## Abstract

Targeting angiogenesis has been considered a promising treatment for a large number of malignancies, including osteosarcoma. Bevacizumab (Bev) is an anti-vascular endothelial growth factor being used for this purpose. We herein investigate the therapeutic potential of Bev in angiogenesis during osteosarcoma and the related mechanisms. Bioinformatics were performed for identification of osteosarcoma-related microarray dataset to collect related lncRNA and miRNA, with MIAT and miR-613 obtained. The predicted binding site between miR-613 and GPR158 3′UTR region was further confirmed by luciferase assay. Then, their effects combined with treatment with Bev on osteosarcoma cells were explored by the gain- and loss-of-function. After extraction from osteosarcoma patients’ serum (serum-EVs) and identification, EVs were co-cultured with osteosarcoma cells, the biological behaviors of which were detected by CCK-8 assay and microtubule formation in vitro. A mouse tumor xenograft model was used to determine the effect of Bev on tumor angiogenesis in vivo. Bev inhibited osteosarcoma cell proliferation and angiogenesis in vivo and in vitro. Besides, serum-EVs could transfer MIAT (EV-MIAT) into osteosarcoma cells, where it is competitively bound to miR-613 to elevate GPR158, thus promoting osteosarcoma cell proliferation and angiogenesis. Furthermore, Bev arrested osteosarcoma cell proliferation and angiogenesis by inhibiting EV-MIAT and inducing miR-613-mediated GPR158 inhibition. In conclusion, the Bev-mediated MIAT/miR-613/GPR158 regulatory feedback revealed a new molecular mechanism in the pathogenesis of osteosarcoma angiogenesis.

## Introduction

Osteosarcoma is the most common malignant bone tumor in children and adolescents, presenting a very heterogeneous genetic feature [1]. Younger age, race, and gender are believed to contribute to higher rates of osteosarcoma [2]. Despite advents in current treatment modalities, including systemic chemotherapy, targeted therapies, immunotherapy, and surgery [3], the overall clinical outcomes of osteosarcoma sufferers remain dismal [4].

Bevacizumab (Bev), a monoclonal antibody against vascular endothelial growth factor (VEGF), has been reported to be the first approved angiogenesis inhibitor and the first available targeted therapy for a range of solid tumors [5]. Injection of Bev in a nude mouse model causes reduced osteosarcoma cell angiogenesis [6]. Angiogenesis is a complicated biological process that plays a key role in osteosarcoma development and progression, and angiogenesis inhibition benefits the prevention of osteosarcoma progression [7]. The serum is an abundant and accessible source of extracellular vesicles (EVs) and serum-derived EVs (serum-EVs) have demonstrated pro-angiogenic effects [8]. EVs are membrane vesicles composed of exosomes and microvesicles, and EV-derived long non-coding RNAs (lncRNAs) exert crucial function in cancer as they are essential for the regulation of hallmarks of cancer, including growth, angiogenesis, and survival of primary tumor, and for the crosstalk between tumor and stromal cells [9]. During the development of osteosarcoma, lncRNA myocardial infarction associated transcript (MIAT) has been identified to be upregulated in tumor tissues, while its knockdown leads to inhibition of osteosarcoma cell proliferation, which was achieved by binding to microRNA-613 (miR-613) [10]. Importantly, osteosarcoma patients present significantly downregulated miR-613 expression, and conversely, elevated expression of miR-613 decreases the proliferation of osteosarcoma cells by directly suppressing CXCR4 [11]. Furthermore, binding sites between miR-613 and GPR158 mRNA were predicted by the starBase database. G-protein-coupled receptors (GPRs) are membrane-bound single peptide chain receptors. As molecular messengers, they transmit extracellular signals to the intracellular region and trigger cellular responses [12]. GPR158 is a newly characterized family GPR and is involved in cognitive processes, glaucoma, and cancers [13]. However, its potential role in bone tumors, including osteosarcoma, is poorly understood. We, therefore, aimed to investigate its role in osteosarcoma in this study and the interaction with the MIAT carried by osteosarcoma patient serum-derived EVs (EV-MIAT)/miR-613/GPR158 axis to participate in the potential molecular mechanism of Bev in angiogenesis during osteosarcoma.

## Materials and methods

### Ethics statement

The current study was implemented with the approval of the Research Ethics Committee of The First Affiliated Hospital of Harbin Medical University and performed in strict accordance with the Declaration of Helsinki. All participants supplied informed consent documentation. Animal experiments were rarified by the Institutional Review Board of The First Affiliated Hospital of Harbin Medical University and performed in light of the Guide for the Care and Use of Laboratory Animals published by the US National Institutes of Health.

### Microarray-based gene expression profiling

Osteosarcoma-related gene expression dataset GSE41445 (three normal samples and three tumor samples) was collected from the Gene Expression Omnibus (GEO) database. Differential analysis was implemented using R language “limma” package with |logFC | > 2 and adj.p.val <0.05 set as the threshold. The false discovery rate (FDR) method was used to correct the difference *p* value. The starBase and RNA22 databases were employed for the prediction of the downstream miRNAs of MIAT. Downstream target genes of miR-613 were predicted using the starBase, miRDB, TargetScan, and mirDIP databases. The binding site between MIAT and miR-613, as well as between miR-613 and GPR158 was predicted using the starBase database.

### Sample collection

Tumor tissues and adjacent normal tissues were surgically obtained from 68 patients with primary osteosarcoma confirmed at The First Affiliated Hospital of Harbin Medical University and Harbin Medical University Cancer Hospital from January 2014 to May 2020. All patients with osteosarcoma were local primary tumors. The clinicopathological characteristics are shown in Supplementary Table 1.

### Cell culture and treatment

Human osteosarcoma cell lines (U2OS and MG63) and 293 T cells were purchased from the Type Culture Collection of the Chinese Academy of Sciences, Shanghai Institute of Cell Biology, Chinese Academy of Sciences. Human umbilical vein endothelial cells (HUVECs) were purchased from Procell Life Science & Technology Co., Ltd. (Wuhan, Hubei, China). Short tandem repeat (STR) analysis and mycoplasma detection were performed in all cell lines used in this study. U2OS, MG63, and HUVECs were cultured in Roswell Park Memorial Institute (RPMI)-1640 medium (Gibco BRL, Rockville, MD, USA). 293 T cells were cultured in Dulbecco’s modified Eagle’s medium (DMEM; HyClone Laboratories, Logan, Utah, USA). The aforementioned mediums were supplemented with 10% fetal bovine serum (FBS; Gibco BR), 100 U/mL penicillin, and 100 μg/mL streptomycin (Invitrogen Inc., Carlsbad, CA, USA), and cell culture was conducted in a 5% CO_2_ incubator at 37 °C.

Cells were transfected with small interfering RNA-negative control (si-NC), si-MIAT, miR-613 mimic, miR-613 inhibitor, mimic NC, and inhibitor NC plasmids (Shanghai GenePharma Co., Ltd., Shanghai, China) using the Lipofectamine 2000 transfection reagent (Life Technologies Limited Paisley, Grand Island, NY, USA). Specific information for the plasmids is shown in Supplementary Table 2. After 48 h of transfection, the cells were cultured in a medium containing puromycin (1 μg/mL) for 2 weeks to select the stably transfected cell lines. The cells were treated with Bev (Sigma-Aldrich, St Louis, MO, USA) at a storage concentration of 25 mg/mL and a final concentration of 1 μg/mL for 24 h.

### Isolation and identification of EVs

ExoQuick kit (System Biosciences, Inc., Mountain View, CA, USA) was used to isolate EVs. Briefly, the serum of patients with osteosarcoma or the medium supernatant secreted by osteosarcoma cells (1 × 10^7^ cells) was collected and centrifuged at 3000×*g* for 15 min. Next, 63 μL of ExoQuick Exosome precipitation solution was added to 250 μL of supernatant, and the mixture was frozen at 4 °C for 30 min. After centrifugation at 1500×*g* for 30 min, 100 μL of EV pellet was resuspended in sterile PBS. Observation of EV morphology was implemented under a transmission electron microscope (TEM; H7650, Hitachi, Japan). The expression of EV-specific markers was detected by means of western blot analysis to identify the characteristics of EVs. Nanoparticle tracking analysis (NTA) with a NanoSight nanoparticle tracking analyzer (Malvern Instruments, Malvern, UK) was utilized to measure the size distribution of EVs.

### Fluorescence microscope for the internalization of EVs by osteosarcoma cells

The EVs were labeled with fluorescent dye PKH67 (Sigma-Aldrich). The osteosarcoma cells expressing PKH67 were placed in the upper chamber of Transwell (Thermo Fisher Scientific Inc., Waltham, MA), and then co-cultured with the osteosarcoma cells that had grown in the lower Transwell chamber for 30 min, 2 h, and 24 h. The osteosarcoma cells in the lower chamber were fixed with 4% paraformaldehyde, and the nuclei were stained with 10 μg/mL 4′,6-diamidino-2-phenylindole (DAPI) staining solution (C1025, Beyotime, Nantong, China) for 10 min. A Nikon Eclipse fluorescence microscope (Nikon, Tokyo, Japan) was used to observe the uptake of labeled EVs by recipient osteosarcoma cells.

### RNA isolation and quantitative real-time polymerase chain reaction (PCR) analyses

The total RNA was extracted from cells and tissues with an AxyPrep Multisource Total RNA Miniprep Kit from Axygen (Corning, NY). The quality and concentration of RNA were determined with NANODROP 2000c Spectrophotometer (Thermo Fisher Scientific). To detect the expression of lncRNA and mRNA, reverse transcription was performed with the help of the Rever TraAce qPCR RT Kit Master Mix with gDNA Remover (FSQ-301, Toyobo Co., Ltd., Osaka, Osaka Prefecture, Japan). To detect the expression of miRNA, reverse transcription was performed by Mir-X™ miRNA First-Strand Synthesis kit (Clontech Laboratories, Mountain View, CA, USA). qRT-PCR was performed on a 7500 FAST Real-Time PCR System (Applied Biosystems) using SYBR Green Assay (Roche Diagnostics GmbH, Indianapolis, IN, USA). LncRNA and mRNA were normalized to GAPDH while miRNA to U6, respectively. The primer sequences are shown in Supplementary Table 3. The fold changes were calculated using relative quantification (the 2^−ΔΔCt^ method).

### Microtubule formation in vitro

Matrigel (BD Biosciences, Franklin Lakes, NJ) was thawed in a 4 °C freezer and the pipette tip, EP tube, and 96-well plate were placed in a −20 °C freezer for precooling. A precooled pipette tip was used to add 200 mL of melted Matrigel to a precooled 96-well plate in an ultra-clean table. The 96-well plate was put in a 4 °C freezer until the liquid level was smooth, then transferred to a 37 °C incubator and left to stand for 2 h to allow solidification. HUVECs were seeded into 96-well plates covered with Matrigel at a density of 2 × 10^4^ cells/well. The suspension was collected from Bev-treated or untreated U2OS or MG63 cells and added to the well. After incubation for 6 h at 37 °C, the structure (tubular) in each well was photographed under a microscope (Leica, Germany), and the angiogenesis plug-in in ImageJ was used to analyze the length of the formed microtubules in each picture.

### Cell counting kit-8 (CCK-8) assay

U2OS and MG63 cells were seeded into a 96-well plate at a density of 3–4 × 10^3^ cells/well. Five repeated wells were set in each group. Next, Bev at a final concentration of 1, 5, and 25 mg/mL was added to the cells. The optical density (OD) value of each well at 450 nm was measured every 24 h on an ELx-800 microplate reader (Biotek Instruments, Winooski, VT, USA) using CCK-8 Kit (Dojindo Laboratories, Kumamoto, Japan).

### Enzyme-linked immunosorbent assay (ELISA)

The conditioned medium supernatant of U2OS or MG63 cells treated or untreated with Bev was collected. ELISA kits (Abcam, Cambridge, UK) were used to detect the levels of HGF (ab100534), VEGF-D (ab233625), VEGF-C (ab9546), VEGF (ab100662), and ANGPTL4 (ab99974). The concentration was determined by measuring the OD value at 450 nm on a microplate reader.

### Dual-luciferase reporter assay

To test the effect of miR-613 on the activity of the GPR158 promoter, the GPR158 regulatory sequence was inserted into the pGL3 promoter vector (wild type [WT]-GPR158). The binding site mutation sequence of GPR158 and miR-613 was constructed and inserted into the pGL3 promoter vector (mutant [MUT]-GPR158). The constructed vectors were co-transfected into U2OS or MG63 cells in 96-well plates with miR-613 mimic or mimic NC using Lipofectamine 2000 reagent (Life Technologies Limited Paisley). The luciferase activity was tested with the Dual-Luciferase Reporter Assay System (Promega Corporation, Madison, WI).

### Western blot analysis

The total protein was extracted from cells or the EVs with enhanced radioimmunoprecipitation assay lysis buffer (Boster Biological Technology Co., Ltd., Wuhan, Hubei, China) with protease inhibitor. After electrophoresis-separation and electro-transfer, the polyvinylidene fluoride membranes were then blocked using 5% bovine serum albumin (BSA) at room temperature for 2 h and underwent overnight incubation at 4 °C with the diluted primary antibodies against GPR158 (ab121388, 1:1000), CD9 (ab223052, 1:1000), CD81 (ab109201, 1:1000), Alix (ab88388, 1:1000), Calnexin (ab22595, 1:1000), and GAPDH (ab8245, 1:5000) as well as with horseradish peroxidase (HRP)-labeled secondary goat anti-nude mouse antibody (ab6808, 1:2000, Abcam). The immunocomplexes on the membrane were visualized using enhanced chemiluminescence reagent (EMD Millipore, Billerica, MA) and band intensities were quantified using ImageJ software, with GAPDH serving as a loading control.

### RNA pull-down assay

Cells were transfected with biotinylated miR-613 (Bio-miR-613), biotinylated miR-613-MUT (Bio-miR-613-MUT), and biotinylated NC (Bio-NC, GenePharma). After 48 h, the cell lysate was incubated with M-280 streptavidin magnetic beads (Invitrogen). The bound RNA was isolated and evaluated by RT-qPCR.

### Orthotopic xenograft model of osteosarcoma in nude mice

Six-week-old BALB/c nude mice (16–18 g; Beijing Vital River Laboratory Animal Technology Co., Ltd., Beijing, China) were housed in a specific pathogen-free environment. The mice were anesthetized by intraperitoneal injection of Sumianxin at a dose of 1 mL/kg. A longitudinal incision of about 0.5 mm in the upper end of the tibia of mouse right hind limb was made under a surgical microscope using microsurgery scissors. The skin was then cut and the soft tissues were carefully separated, with the upper end of the tibia fully exposed. A 1 mL syringe needle was applied to penetrate the bone cavity along the tibia and then was pulled out. Thereafter, 5 μL of MG63 cell suspension (containing 2 × 10^6^ cells/5 μL) stably expressing red fluorescent protein was injected into the tibia by a micro-syringe, followed by sealing with bone wax. The skin was sutured with 5-0 surgical sutures. The entire surgical procedure was completed in an ultra-clean workbench. After inoculation, the tumor growth was observed every day, and the living conditions of the mice were observed and recorded.

On the third day after inoculation, the tumor-bearing nude mice were divided into three groups (*n* = 6): blank (intraperitoneal injection of 0.2 mL sterile normal saline twice a week for 5 weeks), low-dose Bev (intraperitoneal injection of 2 mg/kg Bev [final volume of 0.2 mL] twice a week for 5 weeks), and high-dose Bev (intraperitoneal injection of 5 mg/kg Bev [final volume of 0.2 mL] twice a week for 5 weeks). The general condition of the tumor-bearing mice (including food and water intake, activity response, and mental state as well as their response to external stimuli) was observed daily, with the tumor weighed. All mice were euthanized on the 35th day after tumor inoculation. Tumor growth was monitored and calculated as follows: volume = length × width^2^ × 0.5. Tumors were collected for immunohistochemistry (IHC) staining.

### IHC for microvessel density (MVD)

The osteosarcoma tissue was embedded in paraffin and cut into 5-μm-thick sections. The sections were then dried overnight at 37 °C, dewaxed in xylene, and rehydrated with graded alcohol. Next, the tissue sections were quenched in 3% hydrogen peroxide, blocked with 5% BSA for 1 h, and immunostained with primary antibody against CD31 (ab182981, 1:2000, Abcam) at 4 °C overnight. After PBS washing, the sections were incubated with the secondary antibody for 1 h. All sections were incubated with Envision system-HRP substrate using a Dako REAL Ebision kit (DAKO, Glostrup, Denmark) before observation under a microscope (Leica). Evaluation of MVD in tumor tissues was implemented referring to a previous study [14].

### Statistical analysis

Statistical analysis and mapping were performed using GraphPad Prism 8.0 (GraphPad Software, La Jolla, CA, USA). All experiments were conducted three times independently. The measurement data were described as mean ± standard deviation. Data between two groups were compared using an unpaired *t*-test. One-way ANOVA was used to compare the data of multiple groups and two-way ANOVA was used to analyze the cell proliferation ability at different time points. A value of *p* < 0.05 was statistically significant.

## Results

### Bev inhibits proliferation and angiogenesis of osteosarcoma cells in vitro and in vivo

**Fig. 1:**
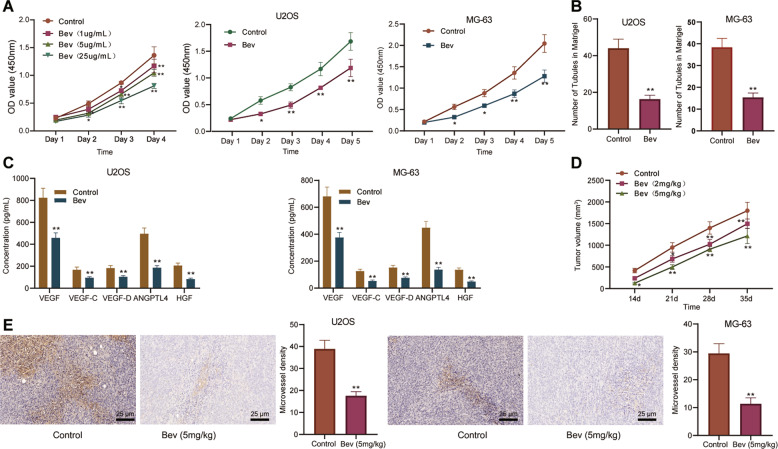
Bev represses the proliferation and angiogenesis of osteosarcoma cells in vitro and in vivo. **A** Proliferation of U2OS and MG63 cells treated with Bev at concentrations of 1, 5, and 25 mg/mL measured by CCK-8 assay. **B** Angiogenesis of Bev-treated U2OS and MG63 cells measured by microtubule formation in vitro. **C** ELISA detection of levels of angiogenesis-related genes HGF, VEGF-D, VEGF-C, VEGF, and ANGPTL4 in the supernatant of Bev-treated U2OS and MG63 cells. **D** Tumor volume of mice treated with Bev. **E** MVD in tumor tissues of mice treated with Bev was detected by IHC (scale bar: 25 μm). *p < 0.05, compared with control cells; **p < 0.01, compared with control mice. *n* = 6 for mice upon each treatment. All experiments were conducted three times independently.

To determine the anti-tumor effect of Bev on osteosarcoma, we firstly used Bev to treat the osteosarcoma cell lines U2OS and MG63. CCK-8 assay results showed that Bev suppressed the proliferation of U2OS and MG63 cells in a concentration-dependent manner (Fig. 1A). The results of microtubule formation in vitro revealed that Bev inhibited the angiogenesis of U2OS and MG63 cells (Fig. 1B). Moreover, ELISA presented lower levels of angiogenesis-related genes HGF, VEGF-D, VEGF-C, VEGF, and ANGPTL4 in the supernatant of Bev-treated U2OS and MG63 cells than those of control cells (Fig. 1C).

Subsequently, we used MG63 cells to construct an orthotopic xenograft model of osteosarcoma in nude mice. The results showed that Bev treatment inhibited the growth of osteosarcoma and decreased tumor volume (Fig. 1D). In addition, IHC exhibited that MVD in tumor tissues was reduced in the presence of Bev (Fig. 1E).

### EV-MIAT promotes osteosarcoma cell proliferation and angiogenesis

**Fig. 2:**
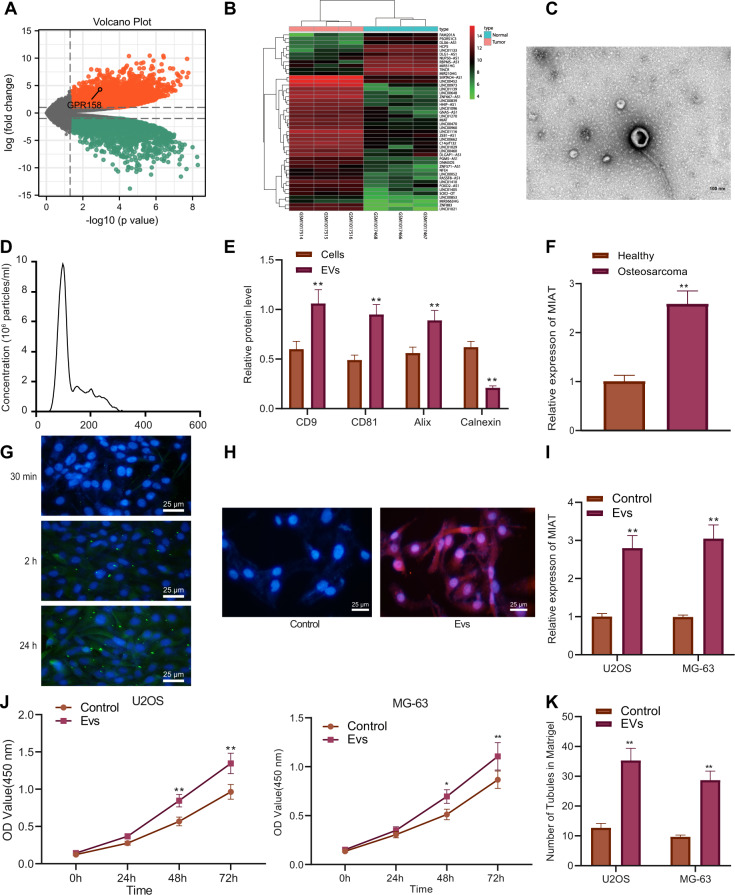
EV-MIAT enhances osteosarcoma cell proliferation and angiogenesis. **A** A volcano plot of differentially expressed mRNAs in osteosarcoma samples from the GSE41445 dataset. The abscissa represents −log10 (*p* value), the ordinate represents logFC, the red dots indicate significantly upregulated genes and the green dots indicate significantly downregulated genes in osteosarcoma samples. **B** A heat map of differentially expressed lncRNAs in osteosarcoma samples from the GSE41445 dataset. The abscissa represents the sample number and the ordinate represents the lncRNA; the left dendrogram refers to the lncRNA expression cluster and the histogram at the upper right refers to a color gradation; each small square in the figure represents the expression of a lncRNA in a sample. **C** Morphological characterization of the serum-EVs observed using a TEM. Scale bar = 100 nm. **D** The size distribution of EVs analyzed by NTA. **E** Western blot analysis of CD9, CD81, Alix, and Calnexin proteins in osteosarcoma cells and the isolated EVs. **F** MIAT expression was determined by RT-qPCR in the serum-EVs from healthy individuals and osteosarcoma patients (*n* = 68). **G** Internalization of EVs by MG63 cells observed under a fluorescence microscope (PKH67-labeled EVs show green fluorescence and DAPI-labeled nucleus show blue fluorescence) (scale bar: 25 μm). **H** Immunofluorescence analysis of Flag-labeled MIAT in MG63 cells following incubation with EVs (Flag-labeled MIAT shows red fluorescence and DAPI-labeled nucleus shows blue fluorescence) (scale bar: 25 μm). **I** MIAT expression was determined by RT-qPCR in U2OS and MG63 cells incubated with EV-MIAT. **J** Proliferation of EV-treated U2OS and MG63 cells measured by CCK-8 assay. **K** Angiogenesis of EV-treated U2OS and MG63 cells measured by microtubule formation in vitro. *p < 0.05, **p < 0.01. All experiments were conducted three times independently.

Next, we shifted our attention to further determine the molecular mechanism by which Bev inhibits the angiogenesis of osteosarcoma. Differential analysis on the GSE41445 dataset yielded 1483 significantly differentially expressed genes in osteosarcoma samples (Fig. 2A). Further screening results showed 46 significantly differentially expressed lncRNAs, among which, MIAT was found to be significantly highly expressed in osteosarcoma samples (Fig. 2B).

The isolated EVs from the serum of patients with osteosarcoma were round or cup-shaped under a TEM (Fig. 2C). NTA results showed that the particle size of EVs ranged between 30 and 300 nm (Fig. 2D). Western blot analysis results displayed that EV markers CD9, CD81, and Alix were not expressed or weakly expressed in osteosarcoma cells, but were highly expressed in EVs, while endoplasmic reticulum membrane protein Calnexin was robustly induced in osteosarcoma cells, but not expressed or expressed very little in EVs (Fig. 2E and Supplementary Fig. 1A). Subsequent RT-qPCR showed that MIAT was abundant in serum-EVs of osteosarcoma patients (Fig. 2F). These results demonstrated the successful isolation of EVs.

In order to explore the effect of EV-MIAT on the proliferation and angiogenesis of osteosarcoma cells, we incubated the isolated EVs with MG63 cells, labeled EVs with PKH67, labeled cell nucleus with DAPI, and observed the uptake of EVs by MG63 cells under a fluorescence microscope. The results showed that the vesicles entered the cell and surrounded the nucleus or were arranged on the inner surface of the cell membrane, and the intake of EVs by MG63 cells was increased significantly over time (Fig. 2G).

Besides, we found the presence of red fluorescence in MG63 cells without MIAT transfection following incubation with EVs for 24 h (Fig. 2H), suggesting that MIAT can be transferred from EVs to MG63 cells. Moreover, RT-qPCR illustrated higher MIAT expression in U2OS and MG63 cells incubated with EV-MIAT relative to the EVs without MIAT (Fig. 2I). At the same time, CCK-8 assay and microtubule formation in vitro showed that treatment with EVs promoted cell proliferation (Fig. 2J) and angiogenesis (Fig. 2K).

### Bev delays the transfer of EV-MIAT to osteosarcoma cells and retards cell proliferation and angiogenesis

**Fig. 3:**
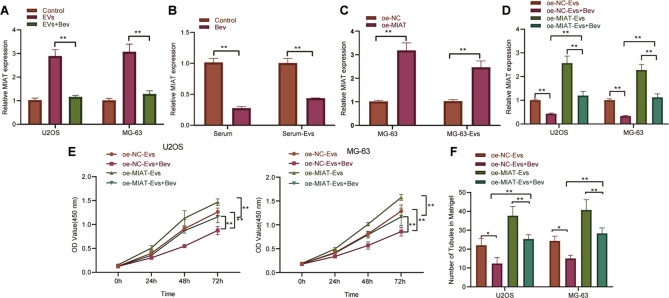
Bev disrupts the delivery of EV-MIAT to osteosarcoma cells to suppress cell proliferation and angiogenesis. **A** MIAT expression was determined by RT-qPCR in U2OS and MG63 cells treated with Bev. **B** MIAT expression determined by RT-qPCR in the serum of mice and serum-EVs following Bev treatment. **C** MIAT overexpression efficiency was determined by RT-qPCR in MG63 cells and MG63 cell-derived EVs. **D** MIAT expression determined by RT-qPCR in U2OS and MG63 cells treated with oe-NC-EVs + Bev, oe-MIAT-EVs, or oe-MIAT-EVs + Bev. **E** Proliferation of U2OS and MG63 cells treated with oe-NC-EVs + Bev, oe-MIAT-EVs, or oe-MIAT-EVs + Bev measured by CCK-8 assay. **F** Angiogenesis of U2OS and MG63 cells treated with oe-NC-EVs + Bev, oe-MIAT-EVs, or oe-MIAT-EVs + Bev measured by microtubule formation in vitro. **p < 0.01. *n* = 6 for mice upon each treatment. All experiments were conducted three times independently.

We then moved to identify whether Bev affects the proliferation and angiogenesis of osteosarcoma cells by regulating EV-MIAT. We found that Bev treatment reduced the expression of MIAT in osteosarcoma cells (Fig. 3A) as well as in the serum of osteosarcoma mice and serum-EVs (Fig. 3B). Meanwhile, MIAT expression was upregulated in MG63 cells and the secreted EVs in the presence of oe-MIAT (Fig. 3C).

RT-qPCR data further revealed that MIAT expression was downregulated in Bev-treated U2OS and MG63 cells that had been incubated with EVs (oe-NC-EVs + Bev), which was negated by further MIAT overexpression (oe-MIAT-EVs + Bev). In addition, treatment with oe-MIAT-EVs + Bev led to a decline of MIAT expression compared to treatment with oe-MIAT-EVs (Fig. 3D).

Additionally, CCK-8 assay and microtubule formation in vitro demonstrated that treatment with oe-NC-EVs + Bev impaired cell proliferation (Fig. 3E) and angiogenesis (Fig. 3F), while a contrary trend was induced following treatment with oe-MIAT-EVs + Bev. Combined treatment with oe-MIAT-EVs and Bev led to decreased cell proliferation and angiogenesis compared to treatment with oe-MIAT-EVs alone. Overall, these findings support that Bev may block the transfer of EV-MIAT to osteosarcoma cells and attenuate the ensuing cell proliferation and angiogenesis.

### EV-MIAT competitively binds to miR-613 in osteosarcoma cells

**Fig. 4:**
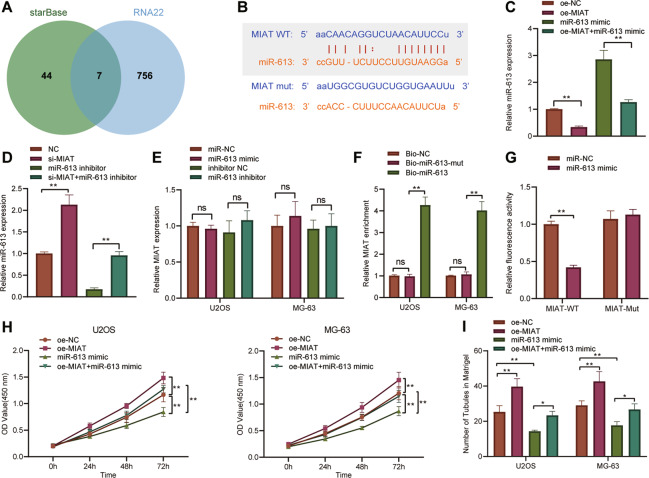
MIAT competitively binds to miR-613, accelerating the proliferation and angiogenesis of osteosarcoma cells. **A** Venn diagram analysis of downstream miRNAs of MIAT predicted by the starBase and RNA22 databases. **B** The predicted binding sites between MIAT and miR-613 by the starBase database. **C** miR-613 expression determined by RT-qPCR in U2OS and MG63 cells transfected with oe-MIAT, miR-613 mimic, or both. **D** miR-613 expression determined by RT-qPCR in U2OS and MG63 cells transfected with si-MIAT, miR-613 inhibitor, or both. **E** MIAT expression determined by RT-qPCR in U2OS and MG63 cells transfected with si-MIAT, miR-613 inhibitor, or both. **F** Enrichment of MIAT in U2OS and MG63 cells transfected with Bio-miR-613 or Bio-miR-613-MUT assessed by RNA pull-down assay. **G** Binding of miR-613 to MIAT confirmed by dual-luciferase reporter assay. **H** Proliferation of U2OS and MG63 cells transfected with oe-MIAT, miR-613 mimic, or both measured by CCK-8 assay. **I** Angiogenesis of U2OS and MG63 cells transfected with oe-MIAT, miR-613 mimic, or both measured by microtubule formation in vitro. *p < 0.05, **p < 0.01. All experiments were conducted three times independently.

The following experiments focused on exploring the molecular mechanism of EV-MIAT in promoting angiogenesis. Intersection analysis of the downstream miRNAs of MIAT predicted by the starBase and RNA22 databases identified seven candidate miRNAs, including hsa-miR-6852-3p, hsa-miR-147a, hsa-miR-4640-5p, hsa-miR-4726-5p, hsa-miR-4766-5p, hsa-miR-613, and hsa-miR-1301-3p (Fig. 4A). The starBase database predicted the binding site between MIAT and miR-613 (Fig. 4B).

Further RT-qPCR results showed that overexpression of MIAT inhibited miR-613 expression while knockdown of MIAT led to an opposite result (Fig. 4C, D). Besides, overexpression or knockdown of miR-613 did not affect MIAT expression (Fig. 4E). RNA pull-down assay exhibited that Bio-miR-613 could enrich MIAT, but Bio-miR-613-MUT failed to enrich MIAT (Fig. 4F). Moreover, luciferase assay indicated that the luciferase activity of MIAT-WT was reduced following transfection with miR-613 mimic (Fig. 4G), suggesting that MIAT can target miR-613 and inhibit miR-613 expression. As depicted in Fig. 4H, I, overexpression of MIAT augmented cell proliferation and angiogenesis, which was inhibited following treatment with miR-613 mimic. Simultaneous overexpression of MIAT and miR-613 led to enhanced cell proliferation and angiogenesis relative to miR-613 overexpression alone. The above data indicate that MIAT might inhibit miR-613 expression by directly binding to miR-613, thus promoting proliferation and angiogenesis of osteosarcoma cells.

### MIAT upregulates GPR158 by competitively binding to miR-613

**Fig. 5:**
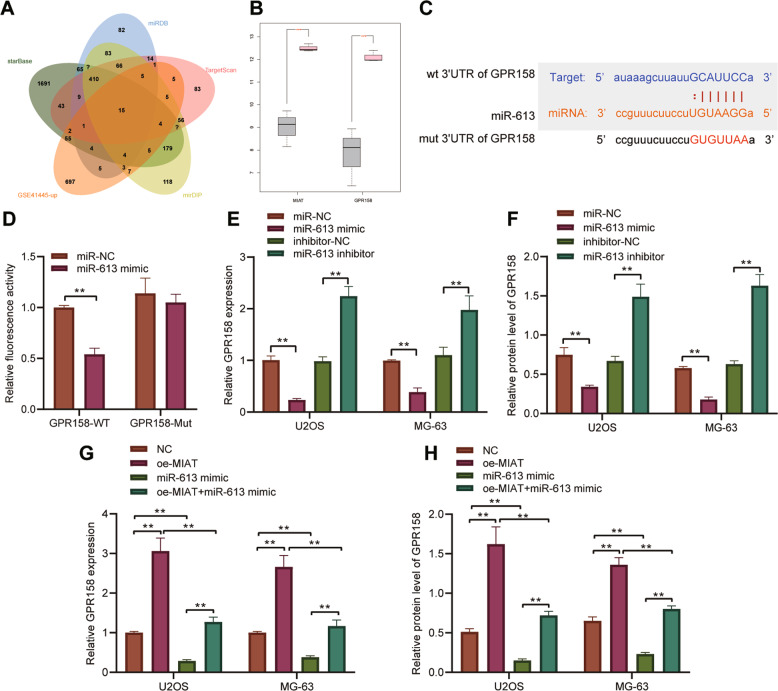
MIAT increases the expression of GPR158 by competitively binding to miR-613. **A** Venn diagram analysis of the predicted target mRNAs of miR-613 by starBase, miRDB, TargetScan, and mirDIP databases, and upregulated mRNAs in the GSE41445 dataset. **B** GPR158 expression in normal and osteosarcoma samples in the GSE41445 dataset. **C** Predicted binding sites between miR-613 and GPR158 by the starBase database. **D** Binding of miR-613 to GPR158 confirmed by dual-luciferase reporter assay in 293 T cells. **E** Expression of GPR158 determined by RT-qPCR in U2OS and MG63 cells transfected with miR-613 mimic or miR-613 inhibitor. **F** Western blot analysis of GPR158 protein in U2OS and MG63 cells transfected with miR-613 mimic or miR-613 inhibitor. **G** Expression of GPR158 determined by RT-qPCR in U2OS and MG63 cells transfected with oe-MIAT, miR-613 mimic, or both. **H** Western blot analysis of GPR158 protein in U2OS and MG63 cells transfected with oe-MIAT, miR-613 mimic, or both. *p < 0.05, **p < 0.01, ***p < 0.001. All experiments were conducted three times independently.

Then we predicted the downstream regulatory mechanism of miR-613. Following Venn diagram analysis of the predicted target mRNAs of miR-613 and those upregulated mRNAs in the GSE41445 dataset, 15 candidate mRNAs were found at the intersection (Fig. 5A). Among the 15 candidate mRNAs, GPR158 was detected to be abundantly expressed in osteosarcoma samples (Fig. 5B).

Prediction results of the starBase database revealed the presence of binding sites between miR-613 and GPR158 (Fig. 5C). Analysis using the dual-luciferase reporter assay further presented that miR-613 mimic decreased the luciferase activity of WT-GPR158 (Fig. 5D). Besides, a decline of GPR158 expression was seen in osteosarcoma cells transfected with miR-613 mimic, whereas a contrary trend was evident upon miR-613 inhibitor treatment (Fig. 5E, F and Supplementary Fig. 1B). Furthermore, as shown in Fig. 5G, H and Supplementary Fig. 1C, GPR158 expression was elevated in oe-MIAT-transfected osteosarcoma cells but was diminished in miR-613 mimic-transfected osteosarcoma cells. Simultaneous overexpression of MIAT and miR-613 led to lower GPR158 expression than MIAT overexpression alone, while higher GPR158 expression was noted in comparison to single miR-613 overexpression.

### Bev delays osteosarcoma angiogenesis by regulating the EV-MIAT/miR-613/GPR158 axis

**Fig. 6:**
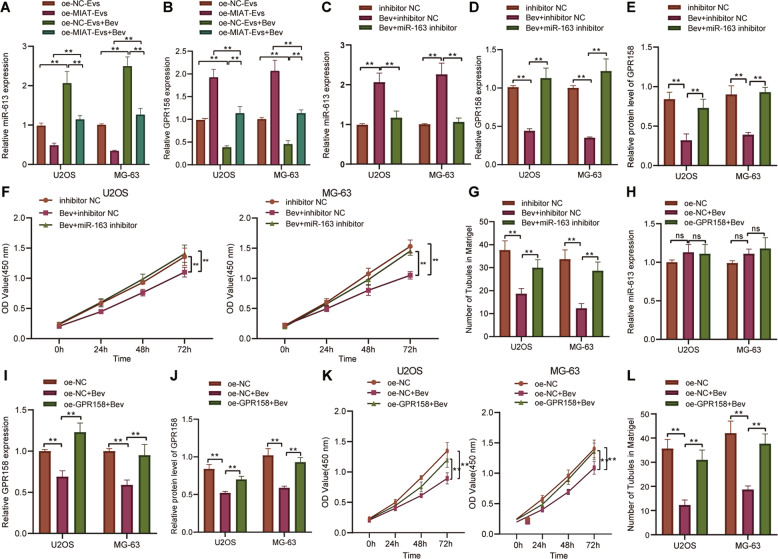
Bev weakens osteosarcoma cell proliferation and angiogenesis by inhibiting EV-MIAT and facilitating miR-613-mediated GPR158 inhibition. **A** miR-613 expression determined by RT-qPCR in U2OS and MG63 cells treated with oe-NC-EVs + Bev, oe-MIAT-EVs, or oe-MIAT-EVs + Bev. **B** GPR158 mRNA expression determined by RT-qPCR in U2OS and MG63 cells treated with oe-NC-EVs + Bev, oe-MIAT-EVs, or oe-MIAT-EVs + Bev. **C** miR-613 expression determined by RT-qPCR in U2OS and MG63 cells treated with Bev + miR-613 inhibitor. **D** GPR158 mRNA expression was determined by RT-qPCR in U2OS and MG63 cells treated with Bev + miR-613 inhibitor. **E** Western blot analysis of GPR158 protein in U2OS and MG63 cells treated with Bev + miR-613 inhibitor. **F** Proliferation of U2OS and MG63 cells treated with Bev + miR-613 inhibitor measured by CCK-8 assay. **G** Angiogenesis of U2OS and MG63 cells treated with Bev + miR-613 inhibitor measured by microtubule formation in vitro. **H** miR-613 expression determined by RT-qPCR in U2OS and MG63 cells treated with oe-GPR158 + Bev. **I** GPR158 mRNA expression determined by RT-qPCR in U2OS and MG63 cells treated with oe-GPR158 + Bev. **J** Western blot analysis of GPR158 protein in U2OS and MG63 cells treated with oe-GPR158 + Bev. **K** Proliferation of U2OS and MG63 cells treated with oe-GPR158 + Bev measured by CCK-8 assay. **L** Angiogenesis of U2OS and MG63 cells treated with oe-GPR158 + Bev measured by microtubule formation in vitro. *p < 0.05, **p < 0.01. All experiments were conducted three times independently.

Finally, we sought to identify the role of Bev in osteosarcoma angiogenesis by regulating the EV-MIAT/miR-613/GPR158 axis. RT-qPCR results indicated that treatment with oe-NC-EVs + Bev upregulated miR-613 expression and diminished GPR158 expression compared to oe-NC-EV treatment alone. Dual treatment with oe-MIAT-EVs + Bev reversed the effect of oe-NC-EVs + Bev on the miR-613 and GPR158 expression. In addition, miR-613 expression was higher while GPR158 expression was lower in response to oe-MIAT-EVs + Bev than oe-MIAT-EVs (Fig. 6A, B).

Furthermore, we discovered an enhancement of miR-613 expression and a decline of GPR158 expression in the presence of Bev + inhibitor NC, while Bev + miR-613 inhibitor negated these trends (Fig. 6C–E and Supplementary Fig. 1D). As illustrated in Fig. 6F, G, Bev + inhibitor NC repressed cell proliferation and angiogenesis, while treatment with Bev + miR-613 inhibitor led to opposite results. This suggests that Bev inhibits osteosarcoma cell proliferation and angiogenesis by elevating miR-613.

Meanwhile, compared with oe-NC-transfected cells, miR-613 expression was enhanced, while GPR158 expression was reduced in the oe-NC-transfected cells treated with Bev (oe-NC + Bev). In the oe-GPR158-transfected cells treated with Bev (oe-GPR158 + Bev), the miR-613 expression did not alter and the mRNA and protein expression of GPR158 was increased compared with those treated with oe-NC + Bev (Fig. 6H–J and Supplementary Fig. 1E). Additionally, the results of CCK-8 assay and microtubule formation in vitro demonstrated that treatment with oe-GPR158 + Bev abolished the inhibiting effect of oe-NC + Bev on cell proliferation (Fig. 6K) and angiogenesis (Fig. 6L). These lines of evidence demonstrate that Bev regulates miR-613-mediated GPR158 by inhibiting EV-MIAT, thus suppressing osteosarcoma cell proliferation and angiogenesis.

## Discussion

Targeting angiogenesis has been considered a promising therapy to treat a large number of malignancies, including osteosarcoma [15, 16]. The summarized findings supported the inhibiting effect of Bev on the angiogenesis of osteosarcoma cells by regulating the EV-MIAT/miR-613/GPR158 axis.

Our initial results provided evidence suggesting that Bev could effectively inhibit the proliferation and angiogenesis of osteosarcoma cells in vitro and in vivo. Bev has been the most extensively well-established anti-angiogenetic treatment option due to its inhibiting effect on the VEGF [5]. In line with our findings, administration of Bev in an osteosarcoma xenograft model in nude mice exhibits strong anti-angiogenesis activity [6]. In addition, Bev has demonstrated anti-proliferation effects on tumor cells like hepatocellular carcinoma cells [17]. It remains to be investigated whether the treatment of Bev would lead to inhibition of proliferation of osteosarcoma cells due to the lack of available literature.

Our study unfolded that the inhibiting effect of Bev on the osteosarcoma cell proliferation and angiogenesis was related to the blockade of transfer of EV-MIAT to osteosarcoma cells. EVs could deliver lncRNAs to the recipient cancer cells, where lncRNAs exerted tumor-promoting effects [18]. Serum EV-MIAT is associated with worse clinical variables and shorter survival of gastric cancer sufferers [19]. Moreover, MIAT is significantly upregulated in both osteosarcoma tissues and cell lines, and this upregulation promotes malignant properties of osteosarcoma cells by enhancing VEGF-C [10]. Therefore, serum-EVs could package MIAT and transferred it to osteosarcoma cells where EV-MIAT can act as a potential driver to stimulate cell proliferation and angiogenesis.

Further analysis exhibited that EV-MIAT is competitively bound to miR-613 in osteosarcoma cells. Consistently, recent literature confirmed the direct binding between MIAT and miR-613 in laryngeal squamous cell carcinoma cells and that MIAT can function as a sponge of miR-613 to downregulate its expression [10]. A previous study confirmed the significant downregulation of miR-613 in osteosarcoma tissues and cell lines, and its elevation in osteosarcoma cells can suppress the osteosarcoma cell malignant behaviors [11]. In the current study, we found that miR-613 could suppress the proliferation and angiogenesis of osteosarcoma cells. Thus, it can be concluded that EV-MIAT can competitively bind to miR-613 in osteosarcoma cells and thus promote osteosarcoma cell proliferation and angiogenesis.

Additionally, MIAT upregulated miR-613 targeted GPR158 by competitively binding to miR-613. Published data have confirmed the miRNA sponge role of lncRNAs and highlighted the implication of the lncRNA/miRNA/mRNA axis in the development of osteosarcoma and providing options for disease diagnosis and treatment [20]. Data from human diseases or dysfunctions identified several GPRs whose mutation was associated with osteosarcoma [21]. PAR1 is a thrombin-responsive GPR and its stimulation causes an enhancement in MALT1 protease activity in both osteosarcoma and breast cancer cells, potentially associating with other aggressive phenotypes [22]. Further, a previously reported study also suggested GPR158 as an important oncogenic factor to promote ovarian carcinoma cell proliferation and progression [23]. We provide the first evidence for the promoting activity of GPR158 in osteosarcoma cell proliferation and angiogenesis, suggesting that GPR158 might be a potential therapeutic strategy for GPR158. Nonetheless, the interaction of the MIAT/miR-613/GPR158 warrants further investigation owing to the little supporting literature elucidating the targeting between MIAT and GPR158, miR-613, and GPR158.

**Fig. 7:**
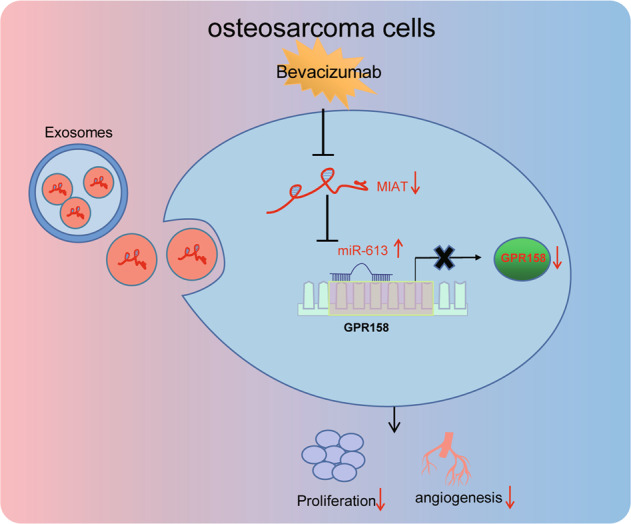
Schematic diagram of the mechanism by which Bev affects osteosarcoma cell proliferation and angiogenesis. Serum-EVs deliver MIAT to osteosarcoma cells. Bev can inhibit the expression of MIAT in osteosarcoma cells and upregulate the expression of miR-613. miR-613 targets GPR158 and inhibits its mRNA and protein expression, thereby inhibiting the proliferation and angiogenesis of osteosarcoma cells.

Overall, our study unveils that Bev can attenuate osteosarcoma cell proliferation and angiogenesis by inhibiting EV-MIAT and facilitating miR-613-mediated GPR158 inhibition (Fig. 7). This novel axis can aid appealing treatment modality concerning angiogenesis in osteosarcoma patients.

## Supplementary information

supplemental materials

aj-checklist

## Data Availability

The datasets generated and/or analyzed during the current study are available from the corresponding author on reasonable request.
