# Supplementary material for: Bevacizumab attenuates osteosarcoma angiogenesis by suppressing MIAT encapsulated by serum-derived extracellular vesicles and facilitating miR-613-mediated GPR158 inhibition
Source: Cell Death Dis. 2022 Mar 28;13(3):272. doi: 10.1038/s41419-022-04620-3 (PMC8960875; doi:10.1038/s41419-022-04620-3)
Supplement: Supplementary file 1 — supplemental materials [file 41419_2022_4620_MOESM1_ESM.docx]

**Supplementary table 1** The clinicopathological characteristics of patients with osteosarcoma.

|  | | Patients | |
| --- | --- | --- | --- |
|  |  | No. | % |
| Sex | male | 44 | 64.71 |
|  | female | 24 | 35.29 |
| Age | ≤14 | 30 | 44.12 |
|  | ＞14 | 38 | 55.88 |
| Subtype | Osteoblastic | 40 | 58.82 |
|  | Chondroblastic | 12 | 17.65 |
|  | other | 16 | 23.53 |
| Location | Femur | 34 | 50.00 |
|  | Tibia | 24 | 35.29 |
|  | Arm | 6 | 8.82 |
|  | Central | 4 | 5.88 |

**Supplementary Table 2** Specific information on the plasmids used for cell transfection

| Group | Intervention |
| --- | --- |
| si-NC | NC of the si-RNA |
| si-MIAT | silencing of MIAT |
| oe-NC | NC of overexpression plasmids |
| oe-MIAT | overexpression of MIAT |
| miR-NC | control of miRNA overexpression |
| miR-613 mimic | overexpression of miR-613 |
| inhibitor NC | control of miRNA inhibition |
| miR-613 inhibitor | inhibition of miR-613 |
| Bio-NC | biotinylated miR-NC plasmid |
| Bio-miR-613-WT | biotinylated miR-613 wild-type plasmid |
| Bio-miR-613-MUT | biotinylated miR-613 mutant plasmid |
| MIAT-WT | MIAT wild-type plasmid |
| MIAT-MUT | MIAT mutant plasmid |
| WT-GPR158 | GPR158 wild-type plasmid |
| MUT-GPR158 | GPR158 mutant plasmid |

**Supplementary Table 3** Primer sequences for reverse transcription quantitative polymerase chain reaction

| Target | Primer sequence |
| --- | --- |
| MIAT (human) | Forward: 5’-GGACGTTCACAACCACACTG-3’ |
|  | Reverse: 5’-TCCCACTTTGGCATTCTAGG-3’ |
| GAPDH (human) | Forward: 5’-AGAAGGCTGGGGCTCATTTG-3’ |
|  | Reverse: 5’-AGGGGCCATCCACAGTCTTC-3’ |
| miR-613 | Forward: 5’-AGGAATGTTCCTTCT-3’ |
|  | Reverse: 5’-GTGCAGGGTCCGAGGT-3’ |
| GPR158 | Forward: 5’-CCCCCAAAGCTGTAGCATCA-3’ |
|  | Reverse: 5’-ATCAGGGCAGACATTGTGGG-3’ |
| U6 | Forward: 5’-GCTTCGGCAGCACATATACTAAAAT-3’ |
|  | Reverse: 5’-CGCTTCACGAATTTGCGTGTCAT-3’ |

**
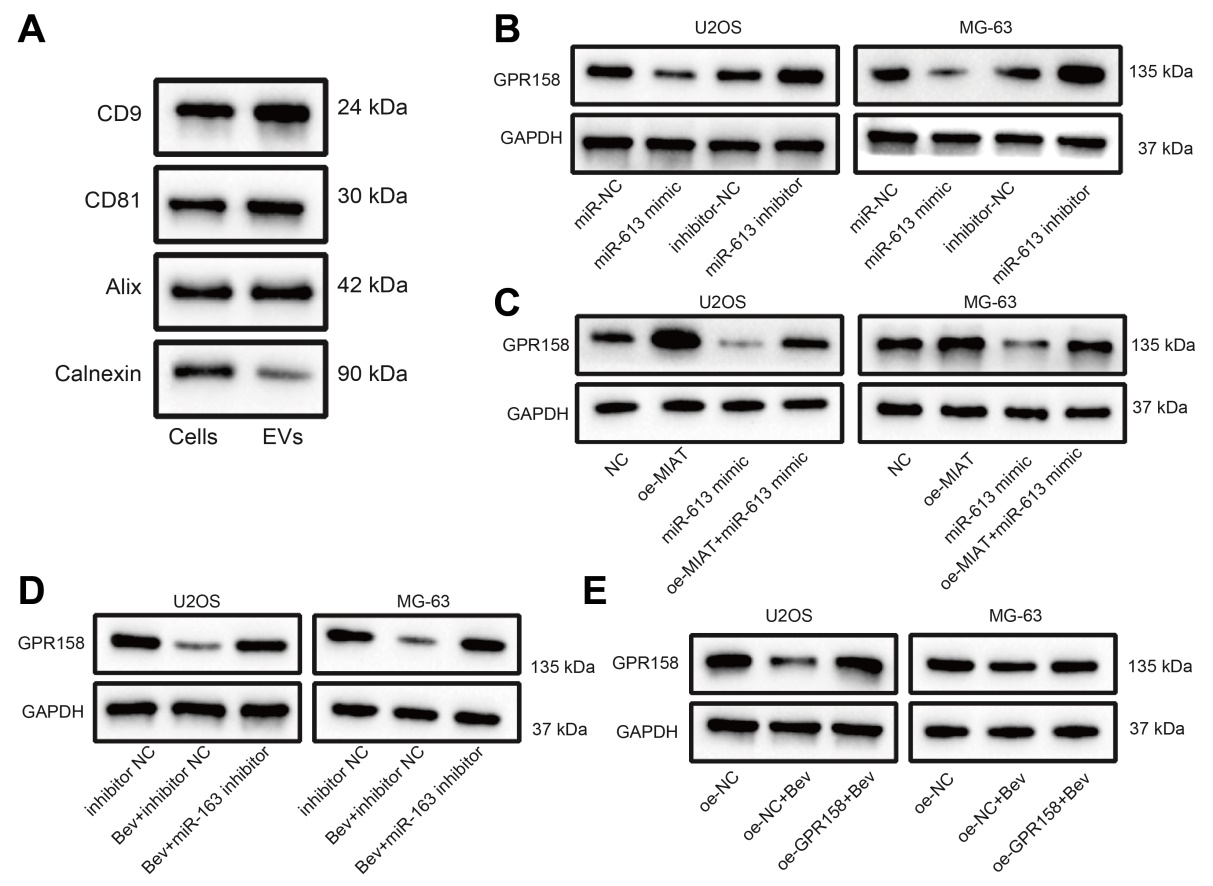
**

**Supplementary Figure 1** Original western blots. A, Western blot analysis of CD9, CD81, Alix, and Calnexin proteins in osteosarcoma cells and the isolated EVs. B, Western blot analysis of GPR158 protein in U2OS and MG63 cells transfected with miR-613 mimic or miR-613 inhibitor. C, Western blot analysis of GPR158 protein in U2OS and MG63 cells transfected with oe-MIAT, miR-613 mimic or both. D, Western blot analysis of GPR158 protein in U2OS and MG63 cells treated with Bev + miR-613 inhibitor. E, Western blot analysis of GPR158 protein in U2OS and MG63 cells treated with oe-GPR158 + Bev.
